# A single nanofluorophore “turn on” probe for highly sensitive visual determination of environmental fluoride ions†

**DOI:** 10.1039/c7ra13601c

**Published:** 2018-02-27

**Authors:** Yangjie Li, Qin Sun, Lei Su, Linlin Yang, Jian Zhang, Liang Yang, Bianhu Liu, Changlong Jiang, Zhongping Zhang

**Affiliations:** Institute of Intelligent Machines, Chinese Academy of Sciences Hefei Anhui 230031 China yangliang@iim.ac.cn zpzhang@iim.ac.cn; Department of Chemistry, University of Science and Technology of China Hefei Anhui 230026 China; State Key Laboratory of Transducer Technology, Chinese Academy of Sciences Hefei Anhui 230031 China; School of Chemistry and Chemical Engineering, Anhui University Hefei 230601 China; Department of Applied Chemistry, Anhui Agricultural of University Hefei Anhui 230036 China

## Abstract

Herein, we report a single nanofluorophore “off–on” probe based on the unique fluoride–boron interaction to achieve the visual determination of fluoride ions in environmental water. Red quantum dots (QDs) were modified using 3-aminophenylboronic acid (APBA) to form a stable standard emission probe, and reaction of the probe with catechol formed a five-membered cyclic borate ester, which led to the quenching of the fluorescence emission. The designed nanofluorophore probe showed a turn-on effect in the presence of fluoride ions due to the five-membered cyclic borate ester being transformed into a trifluoroborate, with breakage of the B–O bonds and removal of the catechol from the QDs. The prepared nanofluorophore probe displayed a high sensitivity for the quantification of fluoride ions with a naked eye visual detection limit of 0.4 μM, which was much lower than the US Environmental Protection Agency (EPA) defined limit (37 μM). Furthermore, the probe displayed an effective application for the detection of fluoride ions in environmental samples such as tap water and lake water. The very simple method reported here could be extended to the visual detection of a wide range of analysis assays in natural samples.

## Abstract 2

A single nanofluorophore “off–on” probe based on the unique fluoride–boron interaction to achieve the highly sensitive visual determination of fluoride ions.

## Graphical abstract

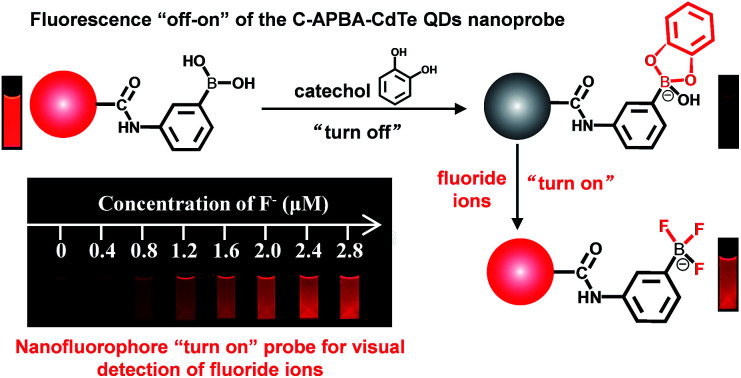

## Introduction

Fluoride is one of the essential trace elements in the human body, and it plays an indispensable role in the environment and in organisms.^1^ A moderate intake of fluoride could prevent dental caries and osteoporosis, thus fluoride ions can be widely found in toothpaste, and it is used to cure bone diseases, but a high concentration is harmful to the human body, which would affect the development of teeth and bones. This would cause the symptoms of chronic fluorosis, like dental fluorosis and skeletal fluorosis, to appear. More seriously, it can cause nausea, vomiting, arrhythmia, and other symptoms of acute fluorosis.^2,3^ In order to prevent such problems, the US Environmental Protection Agency (EPA) and World Health Organization (WHO) have stipulated that the fluoride levels in drinking water should not exceed 37 μM (0.7 ppm) and 79 μM (1.5 ppm), respectively.^4^ Therefore, the sensitive and correct detection of fluoride ions is urgently needed.

There are many conventional analytical techniques for the detection of fluoride ions including ion chromatography (IC),^5^ fluoride ion selective electrodes,^6^ a fluorine reagent colorimetric method,^7^ fluorescence capillary electrophoresis,^8^ and a fluorescent probe method.^9,10^ Fluorescence-based probes have become the most important method for the detection of fluoride ions because of their superior selectivity, high sensitivity, portability, and low cost.^11^ At present, fluorescent probes for fluoride ions can be classified into two types according to their different recognition mechanisms, including a hydrogen bond type and a Lewis acid style.^12–14^ However, hydrogen bonds are not as effective for the detection of fluoride ions because hydrogen bonds can be readily formed between fluoride ions and water molecules, and they are easily interfered with by acetic acid and hydrogen phosphate.^15^ In contrast, Lewis acid style probes can detect an accurate concentration of fluoride ions. A fluorescent probe is a functional structure based on a fluorescent material, like organic dyes, fluorescent proteins, and quantum dots (QDs). Among those probes, QDs, as a zero dimensional nanomaterial, have unique optical properties. Firstly, the optical properties of QDs can be controlled by changing the size and composition of the material.^1^ Secondly, QDs have a wide range of excitation wavelengths, and can be excited by any wavelength spanning from the ultraviolet to the visible light regions. Thirdly, QDs are more stable than organic fluorescent dyes, with fluorescence lifetimes of 100 times longer, and they can undergo repeated excitation for a long time without fluorescence fading and bleaching.^3,4^ Finally, the surface functional groups of the QDs can be controlled qualitatively and semi-quantitatively in the modification process, which improves the predictability and reliability of the experiment.^5–7^

In this paper, we design a fluorescence “off–on” probe based on the unique fluoride–boron interaction^8^ to detect fluoride ions in environmental water. The QDs are connected with catechol *via* the boronic acid, and the formation of a five-membered cyclic borate ester led to the quenching of the fluorescence. In the presence of fluoride ions, the five-membered cyclic borate ester was transformed into a trifluoroborate with breakage of the B–O bonds and removal of catechol from the surface of the QDs and the eventual recovery of the fluorescence. For the design and synthesis of a five-membered cyclic structure for the detection of fluoride ions, 3-aminophenylboronic acid was easily modified with a carboxyl group on the surface of the QDs with the aid of *N*-hydroxysuccinimide (NHS) and *N*-ethyl-*N*′-(3-dimethylaminopropyl)-carbodiimide (EDC). According to previous research,^8^ cyclic esters can be readily formed from boronic acids and diols in aqueous media, thus catechol can react quickly and efficiently with the boronic acid connected on the QD surface, quenching the fluorescence. Owing to the formation of the B–F bonds, the new nanoprobe reported here could selectively and sensitively detect fluoride ions with a naked eye visual detection limit of 0.4 μM in water. In addition, it showed recoveries in the ranges of 96.7–101.8% and 103.6–105.6% for tap water and lake water, respectively, indicating the potential application of the sensor system for the determination of fluoride ions in real environmental samples.

## Experimental

### Reagents and instruments

All chemicals used were of analytical grade. Tellurium powder (Te), sodium borohydride (NaBH_4_), cadmium chloride hydrate (CdCl_2_·2.5H_2_O), potassium fluoride (KF), isopropanol, and acetone were purchased from Sinopharm Chemical Reagent Company, Ltd. (Shanghai, China). 3-Aminophenylboronic acid (APBA), catechol, 3-mercaptopropionic acid (MPA), *N*-hydroxysuccinimide (NHS), and *N*-ethyl-*N*′-(3-dimethylaminopropyl)-carbodiimide (EDC) were purchased from Sigma Chemical Corporation. Aqueous solutions were all prepared using ultrapure water (18.2 MΩ cm) from a Millipore water purification system. All chemicals and solvents were obtained from commercial sources and used directly without further purification, and all glassware was dipped in aqua regia, then cleaned with ultrapure water and dried before use.

Fluorescence spectra were recorded with a Cary Eclipse fluorescence spectrophotometer. The fluorescence emission spectra were recorded in the wavelength range of 560–700 nm with an excitation wavelength of 350 nm, and the slit width was set as 10 nm. In addition, UV absorption spectra were recorded on a Shimadzu UV-2550 spectrometer at room temperature. Infrared spectra were recorded on a Thermo-Fisher Nicolet iS10 FT-IR spectrometer. High-resolution mass spectra (HR-MS) were recorded using an Agilent Q-TOF 6540 mass spectrometer. Solid samples were used to acquire X-ray photoelectron spectra on a model Escalab MK II electron spectrograph (VG). Photographs were taken with a Canon 600D digital camera under an AGL-9406 portable UV lamp (365 nm).

### Synthesis of the carboxyl modified CdTe QDs

The red-emissive CdTe QDs were prepared in aqueous solution according to the reported method with some modifications.^16,17^ Firstly, tellurium powder (0.0638 g) and NaBH_4_ (0.1 g) were dissolved in 2 mL deoxygenated water under a nitrogen atmosphere in an ice bath. When the color gradually changed from black to colorless by stirring for 10 h, the upper transparent clear liquid was sodium tellurite solution (NaHTe). Meanwhile, in another round-bottomed flask, CdCl_2_·2.5H_2_O (0.2284 g) was dissolved in 100 mL ultrapure water and then MPA (0.209 mL) was dropped into the solution. The pH value of the mixture solution was adjusted to 9 by adding 1 M NaOH solution under stirring for at least 30 min to remove the oxygen during this process. Then H_2_SO_4_ (0.5 M, 5 mL) was slowly injected into the prepared NaHTe solution with an injector. The freshly prepared H_2_Te was transferred into the above solution through a rubber hose. Finally, the solution was heated at 110 °C under oxygen-free conditions. The reaction solution was monitored in real-time, until the emission wavelength of the CdTe QDs grew to be centred at 620 nm. Then the reaction solution was cooled down to room temperature. A mixed solvent of isopropanol and acetone (1 : 1) was added to the stock solution to obtain the CdTe QDs as a precipitate. The purified CdTe QDs were dispersed in ultrapure water, and stored at 4 °C in the dark for further use.

The concentration of the CdTe QDs could be calculated using the Beer–Lambert law.*A* = *εCL**A* is the absorbance at the peak position of the first exciton absorption peak for a given sample. As shown in Fig. S1,†*A* is 0.25. *ε* is the extinction coefficient per mol of nanocrystals (L mol^−1^ cm^−1^). According to the literature,^18^*ε* could be calculated by the diameter of the CdTe QDs. *L* is the path length (cm) of the radiation beam used for recording the absorption spectrum. In our experiments, *L* was fixed at 1 cm. Therefore, the concentration of the MPA-CdTe QDs was 1.42 × 10^−6^ mol L^−1^. The concentration was then diluted 2.5 times to subsequently synthesize the 3-aminophenylboronic acid modified CdTe QDs, and this was the final concentration of the probe.

### Preparation of the 3-aminophenylboronic acid modified CdTe QDs (APBA-CdTe QDs)

The CdTe QD solution (4 mL) was mixed with 6 mL phosphate buffer saline (PBS, 10 mM pH = 7.4), then EDC (4 mg) and NHS (4 mg) were introduced into the mixture at room temperature and gently stirred for 30 min to activate the carboxylate groups on the surface of the CdTe QDs. After that, APBA (0.3 mg) was added to the activated CdTe QD solution. After 6 hours of reaction, the APBA-CdTe QDs were washed with an isopropanol and acetone mixed solvent (1 : 1), then dispersed in PBS buffer and stored in the dark.

### Detection of fluoride ions with the nanoprobe

0.15 mg catechol was added to the APBA-CdTe QD solution (10 mL) in PBS buffer under gentle stirring for 4 hours to obtain the nanoprobe solution (C-APBA-CdTe QDs). Then 3 mL C-APBA-CdTe QDs was added into a quartz cuvette for following the detection. The fluorescence emission spectra were recorded under a single excitation wavelength at 350 nm after shaking vigorously for 30 min at room temperature. All the fluorescence intensities were average values of three independent measurements. The color changes were observed under a 365 nm UV lamp.

### Selectivity and interference experiments

To investigate the interference of other anions (Cl^−^, Br^−^, I^−^, HCO_3_^−^, CH_3_COO^−^, HPO_4_^−^, NO_3_^−^, and SO_4_^2−^) on the detection of F^−^, the fluorescence responses of the nanoprobe to these anions were monitored following the same procedure to that mentioned above for F^−^. For the interference study, 28 μM Cl^−^, Br^−^, I^−^, HCO_3_^−^, CH_3_COO^−^, HPO_4_^−^, NO_3_^−^, and SO_4_^2−^ were mixed with the probe. In the case of coexisting anions, then 2.8 μM F^−^ was further added into the probe solution and the fluorescence spectra were collected again.

### Detection of fluoride ions in the tap water and lake water samples

The tap water samples were collected from our lab and the lake water samples were obtained from a local lake. All the water samples were filtered using ordinary qualitative filter paper to remove the macroscopic particulate matters before use. Different concentrations of F^−^ were added into these samples and these treated samples were detected using the nanoprobe solution. The fluorescence emission spectra were recorded and the fluorescence intensity values were average values of three independent measurements.

## Results and discussion

### Synthesis and characterization of the nanofluorophore probe

Surface groups of different types and structures can affect the fluorescence properties of QDs.^19,20^ In this paper, a nanofluorophore “off–on” probe was designed and synthesized to determine fluoride ions based on MPA stabilized CdTe QDs sequentially modified with APBA and catechol. The CdTe QDs were prepared according to previous work.^21,22^ The UV absorption spectra (Fig. S1†) showed that the CdTe QDs had a strong absorbance ranging from 600 nm to 400 nm, and the fluorescence spectra (Fig. S2†) showed that they had an intense emission centred at 620 nm. As depicted in Fig. S3,† the peaks at 2925 cm^−1^, 2845 cm^−1^ and 1410 cm^−1^ were attributed to the C–H bond stretching and deforming vibrations, respectively. The peak at 1560 cm^−1^ is attributed to the characteristic vibration of the C=O bond. In addition, the as-prepared CdTe QDs have an average size of 3 nm and had high crystallinity, as illustrated in Fig. S4.† In short, the CdTe QDs were synthesized successfully.

The CdTe QDs were functionalized with boronic acid groups using APBA prior to quenching their fluorescence with catechol. After modification, the negatively charged carboxyl groups on the surface of the CdTe QDs were changed to strong electron deficient boronic acid groups.^19,23^ The modification process of APBA onto the CdTe QD surface was investigated using XPS studies (Fig. S5†). In detail, the peaks at 399.7 and 402 eV were attributed to the N 1s binding energies of N–C and N–H of the amide groups, respectively. The results of the XPS spectrum confirmed the successful modification with APBA. The values of the UV absorption spectra were similar before and after modifying with APBA (Fig. S1†). However, the fluorescence spectrum had a red shift of 10 nm and consequently was centred at 630 nm after the modification (Fig. S2†), which might arise from a physical size increment of the QDs. The FT-IR spectrum (Fig. S6†) displayed that the new peaks of 1330 cm^−1^, 1230 cm^−1^ and 1072 cm^−1^ were attributed to the characteristic vibration of B–O–H. In addition, the peak at 1600 cm^−1^ revealed the characteristic bond of C=O, and the blue shift must be due to the formation of an amide group between the carboxyl and amino groups.

Catechol can quench the fluorescence of the APBA modified CdTe QDs (APBA-CdTe QDs) *via* the formation of a five-membered cyclic structure between the boronic acid and catechol.

**Fig. 1:**
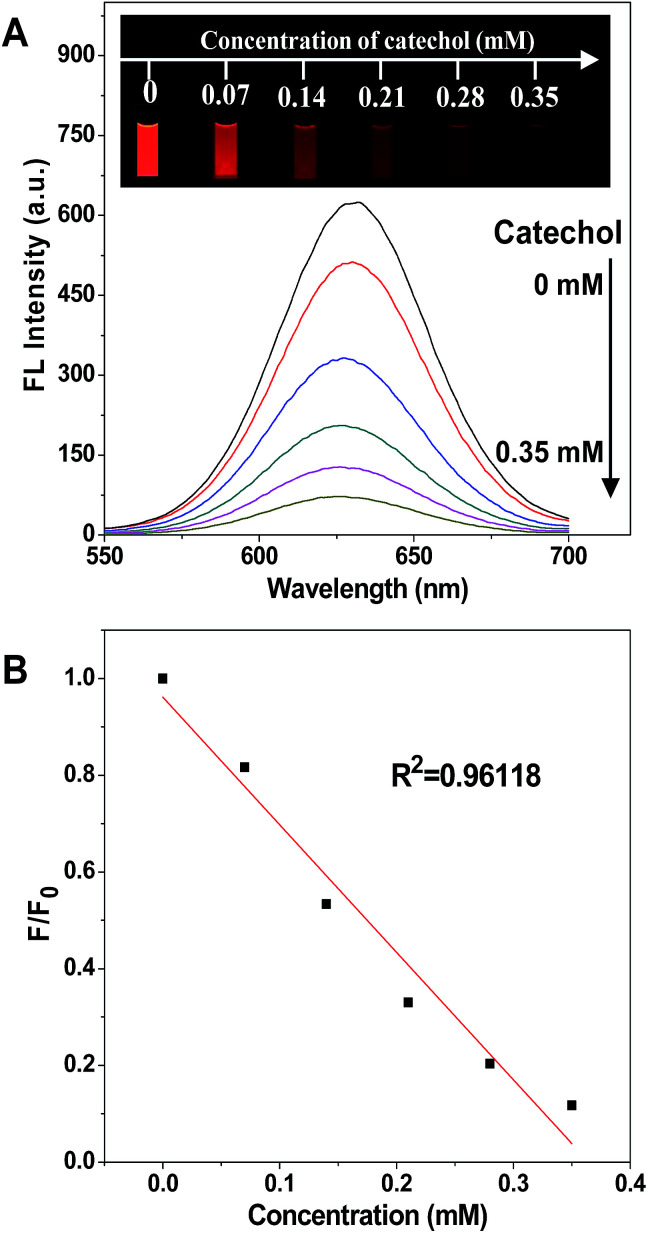
(A) Fluorescence quenching of the APBA-QD solution (4 mL) in PBS buffer with the addition of catechol, and the inset photographs show the fluorescence images of the ABPA-QD solution with different concentrations of catechol under 365 nm UV irradiation. (B) Plot of the fluorescence quenching as a function of the catechol concentration. *F*_0_ and *F* are the fluorescence intensities of the APBA-QD solution in the absence and in the presence of catechol, respectively.

The process of fluorescence quenching is displayed in Fig. 1, showing the intensity of the emission spectrum decreasing with the increase of the catechol concentration, and the fluorescence was nearly quenched when the catechol concentration was 0.35 mM. The photographs shown in the inset, taken under a 365 nm UV lamp, display the red emission of the CdTe QDs fading away on the addition of catechol and eventually turning dark. In contrast, the fluorescence intensity stayed constant while adding the same amount of catechol into an un-modified CdTe QD solution (Fig. S7†), as catechol cannot react with the CdTe QDs directly and so cannot affect their fluorescence properties.

### The mechanism and detection of fluoride ions by the nanofluorophore probe

**Scheme 1:**
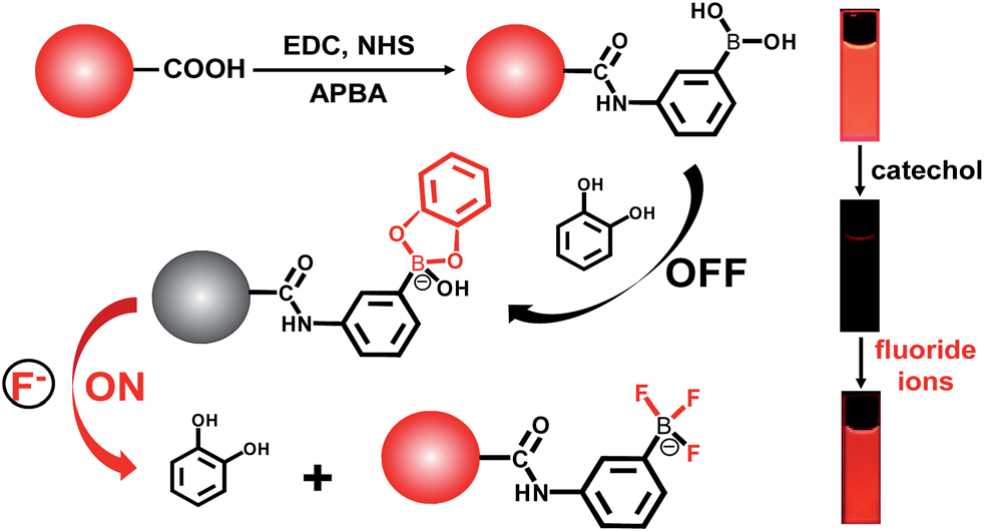
Fluorescence “off–on” mechanism of the C-APBA-CdTe QD nanoprobe for the detection of fluoride ions. The QDs were linked with catechol *via* modification with APBA and the formation of a boronate ester between APBA and catechol, resulting in fluorescence quenching. In the presence of the target F^−^, catechol is removed from the surface of the QDs, leading to the fluorescence recovery of the QDs.

**Fig. 2:**
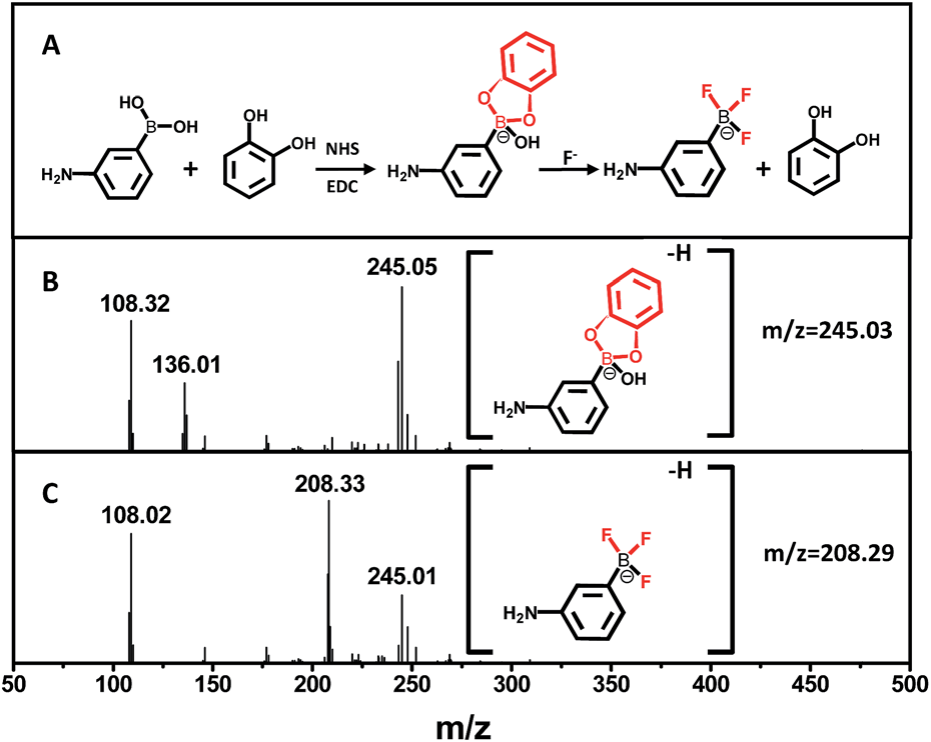
(A) The mechanism of the boronate complex formation and F^−^ detection. (B) HR-MS spectrum of the boronate complex. (C) HR-MS spectrum of the boronate complex after the addition of F^−^.

To determine the presence of fluoride ions (F^−^), the fluorescent probe was designed on the basis of the mechanism that F^−^ could selectively break the chemical bonds between catechol and the boronic acid, which were formed to quench the fluorescence of the APBA-CdTe QDs. The mechanism is depicted in Scheme 1, showing that the CdTe QDs were modified with APBA *via* a commonly used amidation reaction utilizing DSC/NHS as the activator.^21^ After modification, the boronic acid group of the APBA-CdTe QDs reacted with catechol to form a five-membered cyclic structure, which could quench the red emission of the QDs through the formation of a stable boronate complex.^22^ Adding the target analyte fluoride ions into the probe solution led to an obvious recovery of the fluorescence, which was a result of the destruction of the stable five-membered cyclic structure because of the stronger affinity of F^−^ to boron atoms.^24^ In addition, a HR-MS experiment was utilized to verify the feasibility of the detection of fluoride ions, as shown in Fig. 2, which clearly revealed the presence of the reacted products at around *m*/*z* = 245 and *m*/*z* = 208 (Fig. 2B and C). The presence of the five-membered cyclic structure was confirmed by a mass peak of *m*/*z* = 245.03 (calculated *m*/*z* = 246), and its destruction and formation of the trifluoroborate ester (calculated *m*/*z* = 208) were also confirmed by a mass peak of *m*/*z* = 208.29. The results presented clearly verified the mechanism of F^−^ detection.

**Fig. 3:**
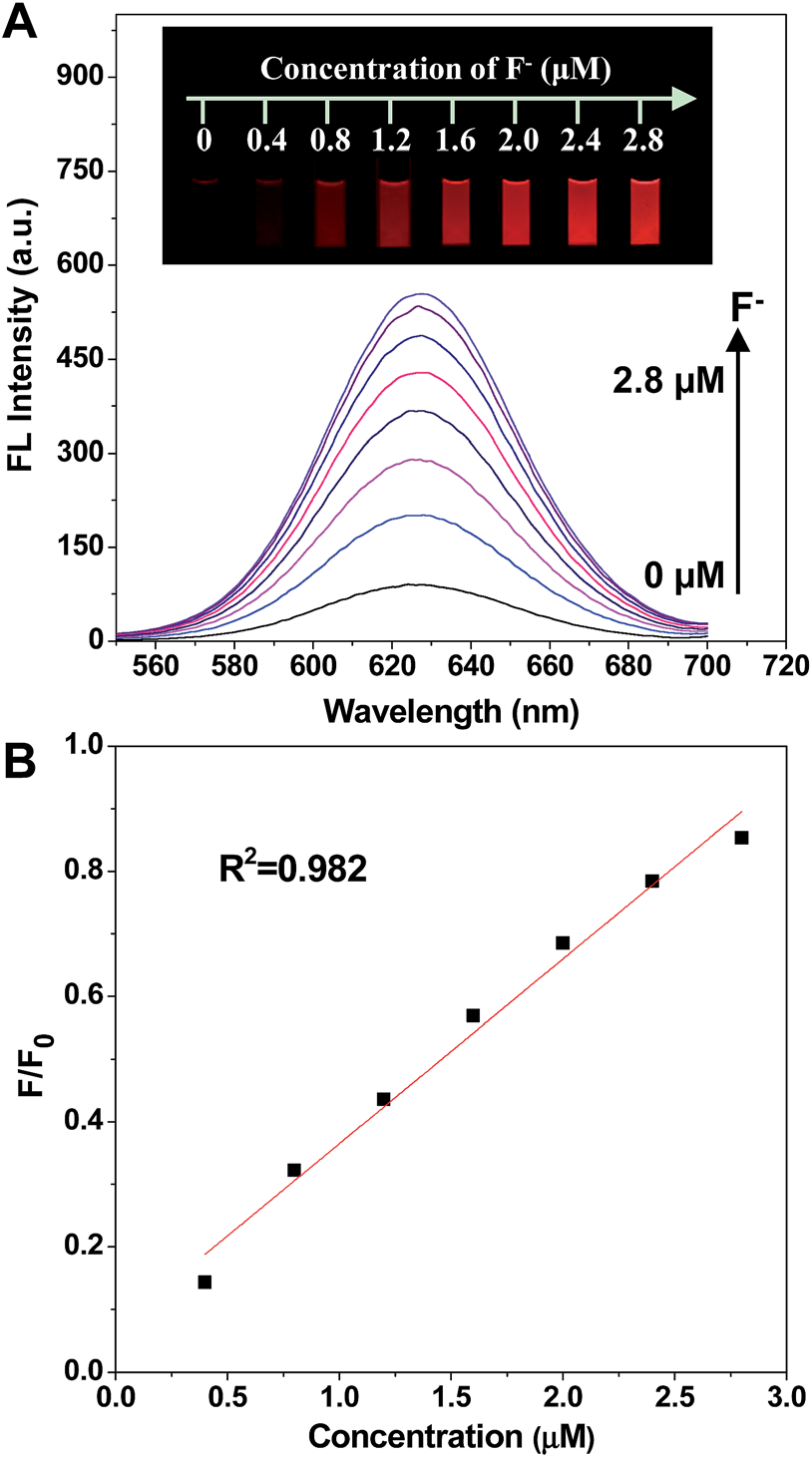
(A) The fluorescence spectra of the C-APBA-CdTe QD solution with the addition of different concentrations of aqueous F^−^, and the inset photographs are the corresponding fluorescence images under a 365 nm UV lamp. (B) The plot displaying the fluorescence recovery ratio of *F*/*F*_0_ as a function of the F^−^ concentration, where *F*_0_ and *F* are the fluorescence intensities of the C-APBA-CdTe QD solution in the absence of and in the presence of F^−^, respectively.

As was expected, the fluorescence intensity was recovered when fluoride ions were added (Fig. 3). The spectra indicated that the fluorescence intensity increased gradually while adding F^−^ in the concentration range of 0.4 to 2.8 μM. Significantly, the fluorescence emission of the prepared nanofluorophore probe had been turned on by F^−^ with a naked eye visual detection limit of 0.4 μM, and the fluorescence of the probe was completely recovered with the addition of 2.8 μM F^−^. Furthermore, the photographs shown in the inset of Fig. 3, taken under a 365 nm UV lamp, illustrate that the recovery of fluorescence leads to a series of lightness changes related to the different F^−^ concentrations, which is of great potential for visual determination. Additionally, the scatter diagram of the relative fluorescence intensity (*F*/*F*_0_) *versus* F^−^ concentration is shown in Fig. 3. In the F^−^ concentration range of 0.4 to 2.8 μM, the linear fitting equation is *y* = 0.29491*x* + 0.06986 (*R*^2^ = 0.982). The prepared nanofluorophore probe displayed a high sensitivity for the quantification of fluoride ions with a naked eye visual detection limit of 0.4 μM, which is much lower than the US Environmental Protection Agency (EPA) defined limit (37 μM) and the World Health Organization (WHO) defined limit (79 μM). This result means that it could be effectively used for applications in the detection of F^−^ in environmental natural samples.

### Selectivity and practical reliability of the probe to fluoride ions

**Fig. 4:**
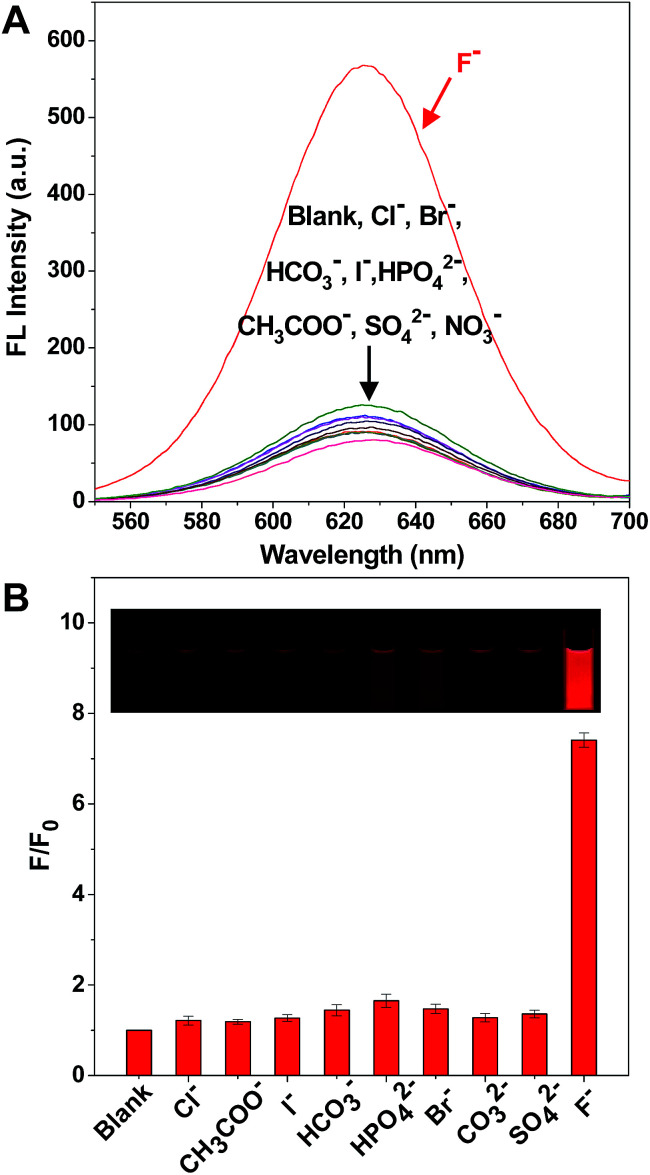
(A) The fluorescence spectra of the C-APBA-CdTe QD probe solution in the presence of 28 μM Cl^−^, Br^−^, I^−^, HCO_3_^−^, AcO^−^, HPO_4_^2−^, NO_3_^−^, and SO_4_^2−^ and 2.8 μM F^−^. (B) The selectivity of the C-APBA-CdTe QD solution with these anions. *F*_0_ and *F* are the fluorescence intensities of the C-APBA-CdTe QD probe solution in the absence and presence of anions, respectively. The inset photographs show the fluorescence changes of the probe solution.

It is worth mentioning that there are a large number of anions in environmental water, which could potentially cause interference in the detection of F^−^. Therefore, it was very significant to test the selectivity of the solution of the C-APBA-CdTe QD probe towards F^−^ by monitoring the response of the fluorescence intensity. Fig. 4A shows the selectivity of F^−^ detection by the C-APBA-QD probe, with the fluorescence intensities of solutions containing 28 μM different anions separately added into the C-APBA-QD solutions under the same conditions, and the corresponding photographs, shown in the inset of Fig. 4B, were taken under a 365 nm UV lamp. The addition of a 28 μM concentration of all the anions, including Cl^−^, Br^−^, I^−^, HCO_3_^−^, CH_3_COO^−^, HPO_4_^−^, NO_3_^−^ and SO_4_^2−^, could not cause a significant recovery of the fluorescence. At the same time, it was easy to distinguish F^−^ from all the other tested anions, but there was no obvious difference in lightness among the other anions under a 365 nm UV lamp. Compared to the blank sample, F^−^ could turn on the fluorescence of the nanoprobe solution with a 7.4 times increase in intensity, while the other anions did not show much of an effect (Fig. 4B). The results here suggested that the probe as-designed could be used for the accurate visual detection of F^−^ with a high selectivity and outstanding sensitivity.

To further examine the practical reliability of the nanofluorophore probe, we applied it to determine F^−^ levels in samples such as tap water and lake water. Fig. 3A was used as the standard color card, and when the concentrations of spiked F^−^ were 1.0, 2.0, and 3.0 μM, it was found that the displayed colors at 1.0, 2.0, and 3.0 μM were almost identical to the standard card, respectively (Fig. S9†). The results confirmed that the as-prepared C-APBA-QD probe can be actually applicable for the detection of F^−^ in water samples. Moreover, we added different amounts of F^−^ into the tap water and lake water samples to test the practicability of this fluorescent probe (Table 1), and the amounts of F^−^ in the samples were measured according to the excellent linear relationship shown in Fig. 3B. The fluorescence recovery values are the ratios of the fluorescence peak intensities on the addition of different amounts of F^−^ in the tap water or lake water samples and the original fluorescence intensity of the APBA-QDs, and were in the ranges of 40–87% and 41–89%, respectively, which indicated the potential application of the as-designed probe for the analysis of F^−^ in real samples. The results of the F^−^ detection further confirmed that the nanoprobe could be actually applied to the sensitive visual detection of F^−^ in environmental water samples.

**Table 1:** The determination of F^−^ spiked in tap water and lake water samples using the nanoprobe

Spiked concentration (μM)	Tap water			Lake water		
	Found (μM)	Fluorescence recovery (%)	RSD (%, *n* = 3)	Found (μM)	Fluorescence recovery (%)	RSD (%, *n* = 3)
1.0	0.967	0.40	2.63	1.056	0.41	3.25
2.0	2.017	0.69	1.84	2.085	0.68	2.87
3.0	3.054	0.87	3.19	3.108	0.89	1.83

## Conclusions

In conclusion, a new fluorescent nanoprobe for the detection of fluoride ions in aqueous solution with the advantages of high sensitivity and selectivity was reported. The nanoprobe was based on MPA-CdTe QDs gradually modified with 3-aminophenylboronic acid (APBA) and catechol. The fluoride ion is one of the strongest Lewis bases, which could react with the boron atom of the borate ester and then break the linkages between APBA and catechol, resulting in the recovery of the fluorescence emission of the quenched QDs. With the increment of the fluoride ion concentration the fluorescence was recovered gradually, and the color changed significantly from dark to red under the excitation of a 365 nm UV lamp. Furthermore, the nanoprobe was applied to determine fluoride ions in environmental samples with satisfying results. The results showed that the nanoprobe reported here could be conveniently and efficiently used in the laboratory and for environmental samples for visual detection.

## Conflicts of interest

There are no conflicts to declare.

## Supplementary Material

RA-008-C7RA13601C-s001
