# Supplementary material for: A single nanofluorophore “turn on” probe for highly sensitive visual determination of environmental fluoride ions
Source: RSC Adv. 2018 Feb 27;8(16):8688–93. doi: 10.1039/c7ra13601c (PMC9082020; doi:10.1039/c7ra13601c)
Supplement: RA-008-C7RA13601C-s001 [file RA-008-C7RA13601C-s001.pdf]

Electronic Supplementary Material (ESI) for RSC Advances.
This journal is © The Royal Society of Chemistry 2018

Electronic Supplementary Information

A single nanofluorophore "turn on" probe for highly sensitive visual determination of environmental fluoride ions

Yangjie Li,‡[a,b] Qin Sun,‡[a,b] Lei Su,[a,b] Linlin Yang,[a,c] Jian Zhang,[a,e] Liang Yang,*[a,c] Bianhu Liu,[a,c] Changlong Jiang[a,c] and Zhongping Zhang*[a,c,d]

[a] CAS Center for Excellence in Nanoscience, Institute of Intelligent Machines, Chinese Academy of Sciences, Hefei, Anhui 230031, China.

[b] Department of Chemistry, University of Science and Technology of China, Hefei, Anhui 230026, China.

[c] State Key Laboratory of Transducer Technology Chinese Academy of Sciences, Hefei, Anhui, 230031, China.

[d] School of Chemistry and Chemical Engineering, Anhui University, Hefei, Anhui 230601 China.

[e] Department of Applied Chemistry, Anhui Agricultural of University, Hefei, Anhui 230036, China.

*Corresponding author E-mail: yangliang@iim.ac.cn, zpzhang@iim.ac.cn.

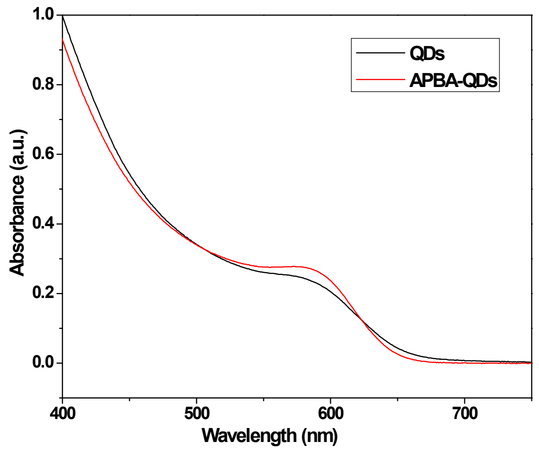

Fig. S1 Absorption spectra of CdTe QDs (black) and APBA-QDs (red). Solution were prepared in 10 mM PBS buffer (pH=7.4).

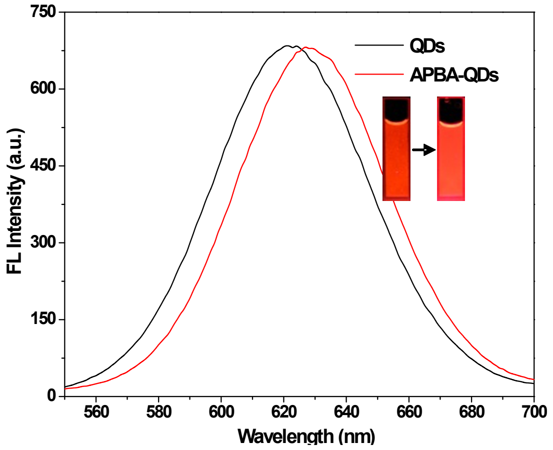

Fig. S2 The fluorescence emission spectra of CdTe QDs (black) and APBA-QDs (red). Inset photographs of CdTe QDs (left) and APBA modified QDs (right) in aqueous solution were taken under a 365 nm UV lamp.

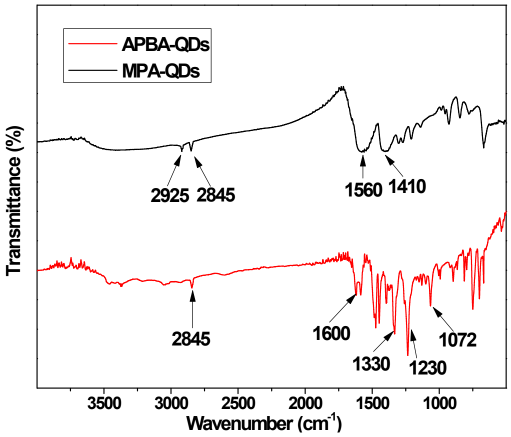

Fig. S3 The FT-IR spectra of MPA-QDs (black) and APBA-QDs (red).

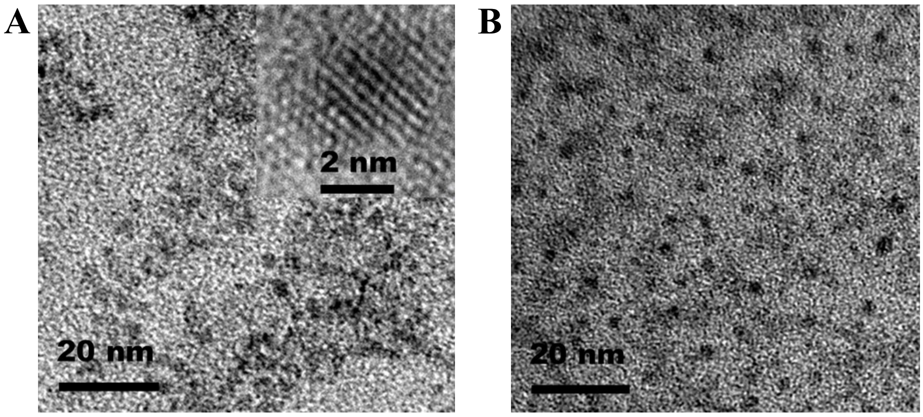

Fig. S4 TEM images of (A) the QDs (inset: the corresponding HR-TEM image) and (B) the modified completely nanoprobe.

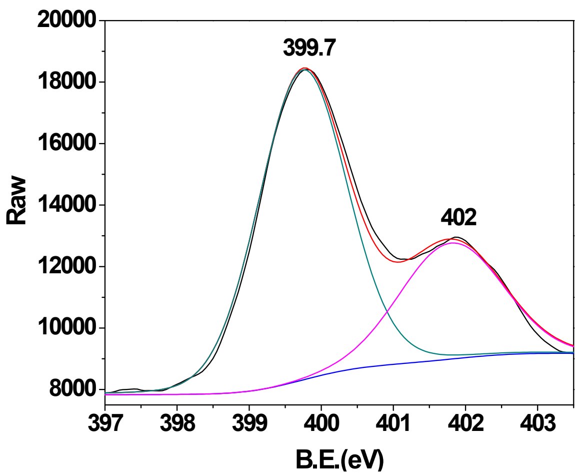

Fig. S5 XPS spectrum of the as-prepared APBA modified CdTe QDs. In detail, 399.7 and 402 eV were attributed to N 1s binding energies of N-C and N-H of amide groups, respectively.

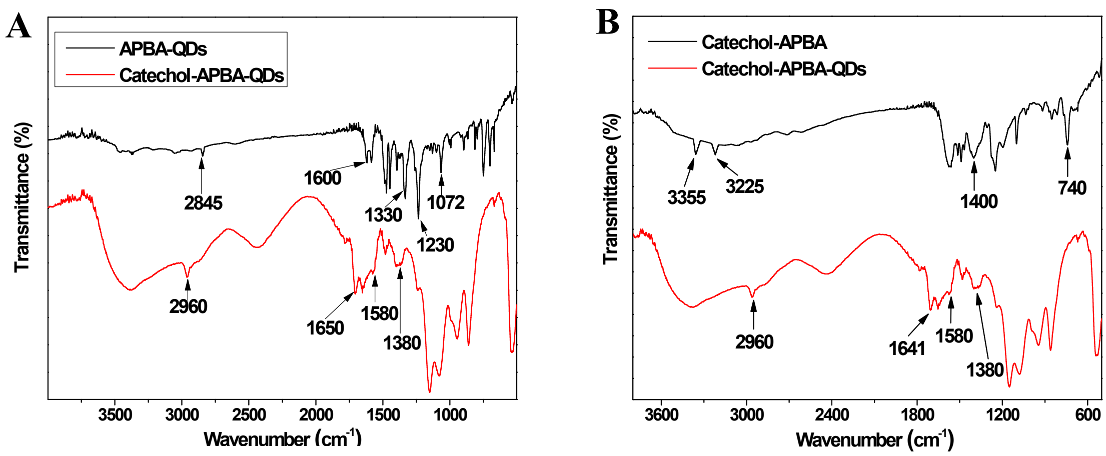

Fig. S6 (A) The FR-IT spectrum of APBA-QDs (black) and catechol-APBA-QDs (red). (B) The FR-IT spectrum of catechol-APBA (black) and catechol-APBA-QDs (red).

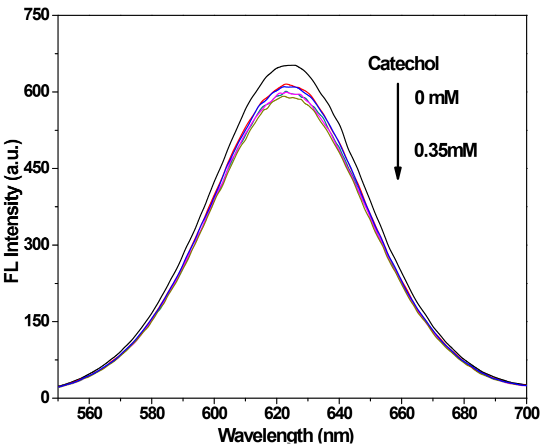

Fig. S7 The fluorescence spectra of the unmodified CdTe QDs solution with the addition of catechol.

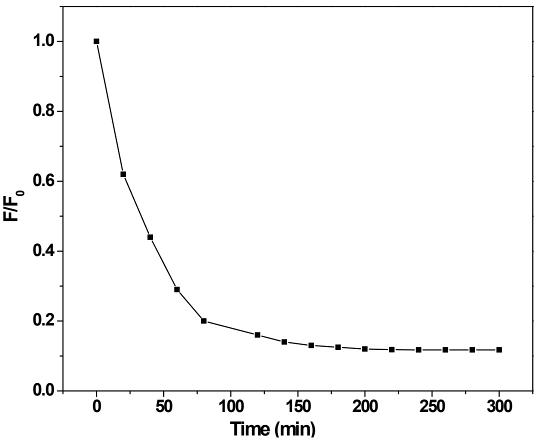

Fig. S8 The kinetics of fluorescence quenching of the APBA-QDs aqueous solution. The fluorescence quenching efficiency (F/F_0) of APBA-QDs via time with addition of 0.35 mM catechol (relative fluorescence intensities are represented as F/F_0, while F_0 and F correspond to the fluorescence intensity before and after the addition of catechol, respectively).

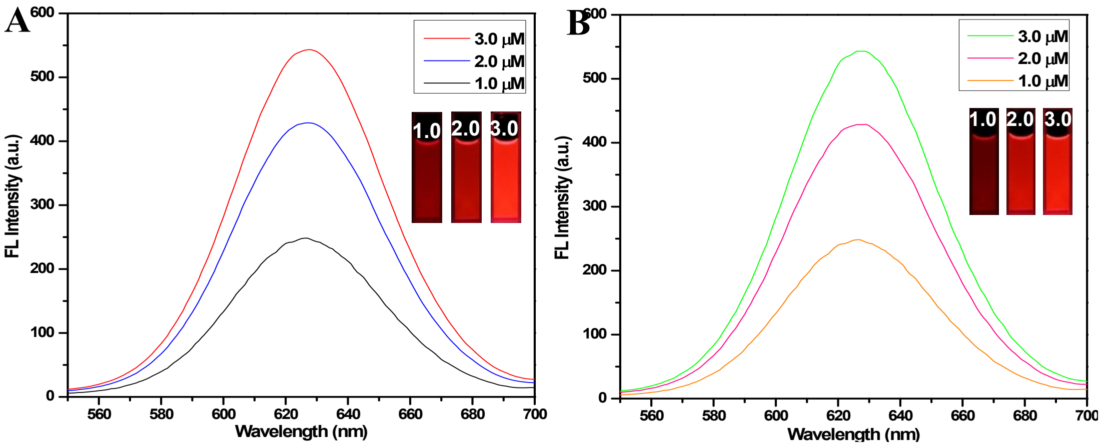

Fig. S9 The fluorescence spectra of the nanoprobe with the addition of the lap water (A) and lake water (B) which spiked with F^- (1.0 μM, 2.0 μM, 3.0 μM). The inset photographs, corresponding to the three different concentrations of F^-, were taken under a 365 nm UV lamp.
